# The effects of nalmefene on the impulsive and reflective system in alcohol use disorder: A resting-state fMRI study

**DOI:** 10.1007/s00213-022-06137-1

**Published:** 2022-04-15

**Authors:** Nadja Grundinger, Sarah Gerhardt, Damian Karl, Karl Mann, Falk Kiefer, Sabine Vollstädt-Klein

**Affiliations:** 1 Department of Addictive Behavior and Addiction Medicine, Central Institute of Mental Health, University of Heidelberg, Medical Faculty Mannheim, PO Box 12 21 20, 68072 Mannheim, Germany; 2 Feuerlein Center On Translational Addiction Medicine (FCTS), University of Heidelberg, Heidelberg, Germany; 3 Mannheim Center for Translational Neurosciences (MCTN), Medical Faculty of Mannheim, University of Heidelberg, Mannheim, Germany

**Keywords:** Alcohol use disorder, Pharmacotherapy, Nalmefene, Resting-state functional connectivity, Impulsive system, Reflective system, Salience network, Reduced drinking

## Abstract

**Rationale:**

Central aspects of alcohol use disorder (AUD) are the irresistible desire for alcohol and impaired control over its intake. According to the triadic neurocognitive model of addiction, this arises from aberrant functioning of different neural and cognitive systems: an impulsive system, a reflective system, and the abnormal dynamics between both systems based on an insular-dependent system.

**Objectives:**

In this study, we examined the effects of a single dose of nalmefene on resting-state functional connectivity (rsFC) patterns within and between these addiction-related neural systems in AUD.

**Methods:**

Non-treatment seeking participants with AUD (*N* = 17; 19–66 years, 6 female) took part in a randomized, placebo-controlled, double-blind, crossover study and received either a single dose of 18 mg nalmefene or a placebo. Using seed-based correlation analyses on resting‐state functional magnetic resonance imaging data, we examined the effects of nalmefene on key nodes related to the (1) impulsive system; (2) reflective system; (3) salience network; and (4) default mode network.

**Results:**

Under nalmefene, participants showed reduced rsFC between components of the impulsive system (Nucleus accumbens–putamen/pallidum/insula). Reduced rsFC was found between elements of the reflective system and impulsive system (orbitofrontal cortex–insula/putamen/pallidum), salience network (orbitofrontal cortex–insula/inferior frontal gyrus), and default mode network (lateral prefrontal cortex–precuneus/cuneus). Components of the salience network showed both increased (anterior cingulate cortex) and decreased (insular cortex) rsFC to elements of the reflective system.

**Conclusion:**

A single dose of nalmefene impacts rsFC and alters the interaction between key nodes of addiction-related neural systems in non-treatment seeking participants with AUD. Nalmefene may normalize rsFC patterns by weakening the impulsive system while strengthening the reflective system.

Trial registration: clinicaltrials.gov: NCT02372318.

**Supplementary Information:**

The online version contains supplementary material available at 10.1007/s00213-022-06137-1.

## Introduction

Alcohol is associated with a multitude of social- and health-related damages (Lim et al. 2012; Shield et al. 2020) that result in high alcohol-related morbidity and mortality (Kraus et al. 2015). Nevertheless, health-care studies show that many patients with alcohol use disorder (AUD), who require treatment, do not receive appropriate therapy (Rehm et al. 2015; Hasin and Grant 2015). One reason for this treatment gap may be the therapeutic goal of life-long abstinence—a feat that is unattainable for many patients (SAMHSA 2014). An alternative could be the targeted reduction of alcohol consumption (Mann et al. 2017; Henssler et al. 2021) with the pharmacological support of the opioid receptor antagonist nalmefene (Selincro®, H. Lundbeck A/S, Valby, Denmark).

In the course of AUD, the initial hedonic effects diminish while consumption becomes increasingly habitual and ultimately compulsive (Volkow et al. 2016). Resting-state functional connectivity (rsFC) can provide new insights into aberrant functioning of the neural circuits of the brain. It allows the identification of functional connectivity (FC) patterns at rest, i.e., consistent patterns of organized and continuous intrinsic activity over space and time (Biswal et al. 1995; Fox et al. 2005; Friston 1994).

Individuals with AUD show network-specific anomalies in their FC patterns, which differ from healthy controls (HC) in spatial extent and strength, as well as in their dynamic interaction with other networks (Müller-Oehring et al. 2015). Thus, AUD can be associated with dysregulation of the interaction between and within well-defined brain networks, such as the Default Mode Network (DMN), the Reward Network (RN), the Executive Control Network (ECN), and the Salience Network (SN; Sutherland et al. 2012; Camchong et al. 2013a; Weiland et al. 2014; Kohno et al. 2017; Chanraud et al. 2011; Fede et al. 2019). Taken together, AUD appears to cause an imbalance between the neural systems for reward and those of cognitive control, possibly leading to deficits in the regulation of craving and, thus, contributing to relapses (Kohno et al. 2017).

According to the triadic neurocognitive model of addiction (Noël et al. 2013), “weak willpower” associated with drug addiction can be described by abnormal functioning of three neural and cognitive systems: (1) an impulsive system, which mediates habitual and automatic behavior; (2) a reflective system, which is important for self-regulation, impulse control, and decision-making; and (3) an insular-dependent system, which translates interoceptive signals into conscious emotional states (such as craving), and in turn decisively modulates the dynamics between the other two systems. In this regard, Zhu and colleagues investigated rsFC in patients with AUD as compared to HC in a model-free approach. They identified differential connectivity patterns within and between resting-state networks (RSN) associated with the triadic neurocognitive model of addiction: an amygdala–striatum network, as the impulsive system; the ECN and orbitofrontal cortex network (OFCN), summarized as the reflective system; the SN involving the insula; and the DMN (Zhu et al. 2017).

Additionally, several other studies investigate how altered rsFC can be interpreted in terms of treatment success. A review by Wilcox and colleagues shows that increasing connectivity within the ECN and between the ECN and SN, as well as higher anti-correlation between ECN and DMN, leads to better treatment outcomes in substance use disorders (SUD; Wilcox et al. 2019). Patients with AUD, who have not successfully completed treatment, displayed greater rsFC between striatum–insula, ECN–amygdala, and SN–striatum/insula/precuneus as well as weaker frontostriatal connectivity between striatum and dorsolateral prefrontal cortex (dlPFC) compared to completers. Interestingly, for AUD patients who did not complete treatment, increased craving is associated with striato-limbic rsFC. In completers, on the other hand, craving is associated with cortico-striatal rsFC (Kohno et al. 2017). Furthermore, long-term abstainers show progressively higher resting-state synchronicity within ECN, which is associated with improved cognitive flexibility, whereas synchronicity decreases within the RN (Camchong et al. 2013b). Altered FC patterns may be seen as an important treatment goal that could reduce the risk of relapse and improve clinical outcomes. Thus, relapse-prevention agents could be used to restore neural circuit function. However, there are few studies to date that investigate the effects of treatment on rsFC (Wilcox et al. 2019).

Nalmefene is used as medication on demand in the treatment of AUD to reduce alcohol consumption. It binds selectively to opioid receptors, whereby an antagonistic effect at the μ- and δ-receptor has been demonstrated, as well as a partial agonistic effect at the κ-opioid-receptor (Bart et al. 2005). Endogenous opioids released by alcohol are directly and indirectly involved in the modulation of mesolimbic dopaminergic activity. Accordingly, dopamine activity in the Nacc is directly regulated by δ-opioid receptors. In addition, stimulation of μ-opioid receptors suppresses inhibition of GABA interneurons, which indirectly triggers dopamine release in the ventral striatum (Devine et al. 1993). κ-opioid receptors (or dynorphin system) also regulate dopamine release through their direct action on dopamine neurons but are associated with the negative components of drug use (Koob 2009; Ciccocioppo 2002). Thus, nalmefene could potentially help rebalance the dysfunctional reward system through its modulatory effect on opioid receptors.

In fact, a study on non-treatment seeking individuals with AUD showed that a single dose of nalmefene significantly reduced neural activity in the striatum during monetary reward anticipation under the influence of alcohol (Quelch et al. 2017). In a recent study by Karl et al. (2021), nalmefene appeared to reduce reactivity to alcohol stimuli in the ventral but not the dorsal striatum, which was associated with significantly lower self-reported craving. In addition, nalmefene seemed to increase neural activity in brain regions mainly associated with empathy and social cognition in individuals with AUD (Vollstädt-Klein et al. 2019). To the best of our knowledge, no study exists, which investigates the influence of nalmefene on rsFC in non-treatment seeking individuals with AUD.

Therefore, we seek to address this gap by investigating whether nalmefene leads to changes in rsFC in neural systems associated with “weakened willpower” in the context of AUD. In this regard, we concentrate on the neural systems of the triadic neurocognitive model of addiction (Noël et al. 2013) and the RSNs found in the study by Zhu et al. (2017). The aim of this work was to use seed-based correlation analysis (SCA) to investigate rsFC patterns under 18 mg nalmefene between components of the following addiction-related networks: (1) Impulsive System–Nucleus accumbens (Nacc); (2) Reflective System–lateral prefrontal cortex (lPFC) and orbitofrontal cortex (OFC); (3) Salience Network–insular cortex and anterior cingulate cortex (ACC); and (4) Default Mode Network–medial prefrontal cortex (mPFC) and posterior cingulate cortex (PCC).

## Methods

The Ethics Committee of the Medical Faculty Mannheim at the University of Heidelberg, Germany, approved this study (registration at clinicaltrials.gov; NCT02372318). All clinical trials were conducted in accordance with the Declaration of Helsinki.

The target group consisted of non-treatment seeking participants between 18 and 70 years with a diagnosis of AUD according to the Diagnostic Statistical Manual of Mental Disorders (DSM-5; American Psychiatric Association 2013). This meets the criteria of the dated term “dependence” (Dawson et al. 2013) and those of the ICD-10. Only heavy drinkers were considered (< 60 g alcohol/day for men; < 40 g alcohol/day for women; at least 5 days per week). A detailed listing of all inclusion and exclusion criteria can be found in the Supplementary Information (Table S1).

**Fig. 1:**
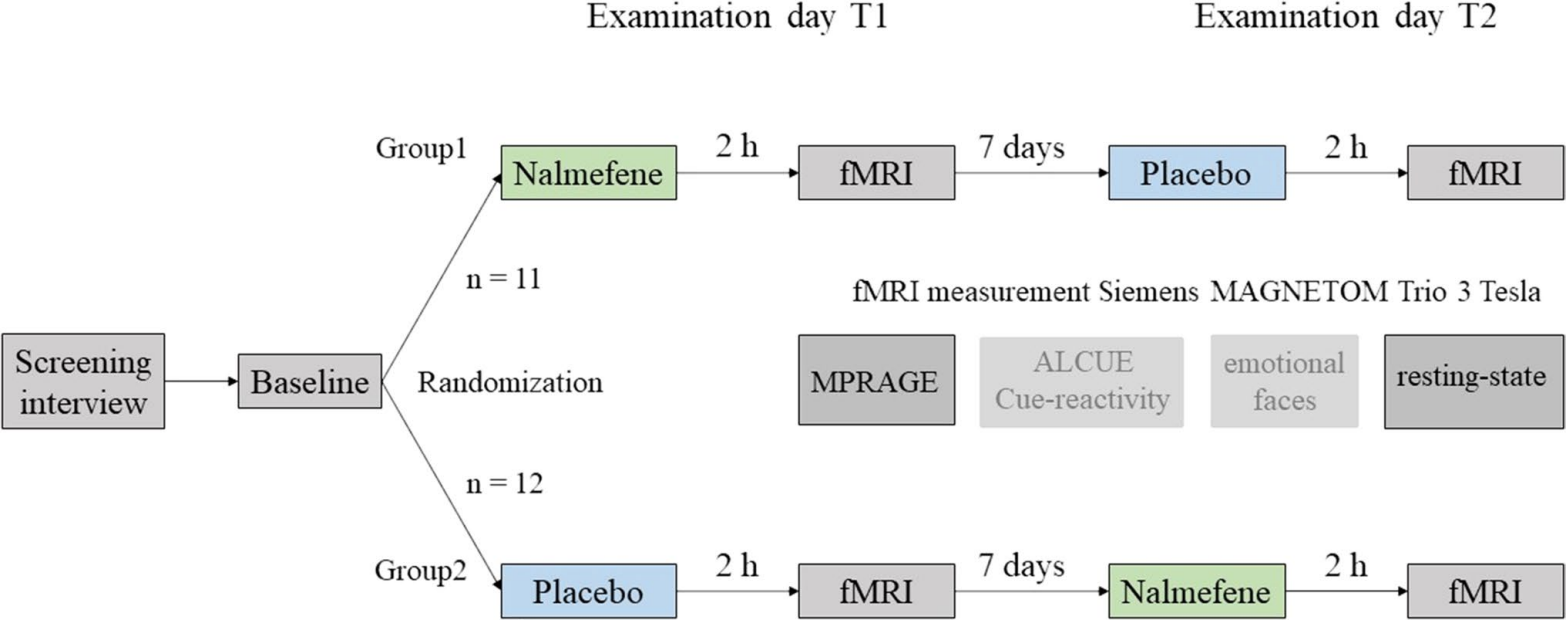
Detailed overview of study design and procedure. During fMRI measurement, participants worked on a cue-reactivity task for alcohol-associated stimuli (Karl et al. 2021) and an emotional faces processing task (Vollstädt-Klein et al. 2019)

The study was conducted as placebo-controlled, double-blind, crossover design. A sample size of 15 participants was estimated to be sufficient to detect a large effect size of f = 0.4 (nalmefene vs. placebo) with at least 80% power (repeated measures ANOVA within factors, estimation with the software G*Power [http://www.gpower.hhu.de]). Due to the double-blind study design, the randomization plan was prepared externally in advance for a total of 40 subjects (to ensure sufficient size in case of dropouts etc.) and consisted of blocks of four, resulting in ten blocks. At the baseline screening, participants were informed in detail about the study procedure and possible risks of the fMRI examination or possible side effects of the drug. Participants signed a written declaration of consent. Sociodemographic data, as well as history of somatic illnesses, mental, or neurological disorders, and current medication, was recorded. In addition to a medical examination (alcohol breath test, urine test for drugs or pregnancy), various neurological tests, questionnaires, and interviews were conducted. The interval between the two examination days, T1 and T2, was 1 week. After a medical examination and review of all inclusion and exclusion criteria, the study medication (nalmefene or placebo) was administered orally. With this form of administration, nalmefene reaches the highest plasma concentration after about 1 to 1.5 h (Kyhl et al. 2016). fMRI measurement was performed 2 h after administration. During resting-state, the participants were instructed to close their eyes without falling asleep, not to think of anything specific, and to let their thoughts wander. After a final medical check-up, participants were discharged. Please see Fig. 1 for details.

### *fMRI acquisition*

The scan was conducted on a Siemens MAGNETOM 3 Tesla whole-body-tomograph (MAGNETOM Trio, TIM technology, Siemens, Erlangen, Germany) equipped with a 12-channel head coil. For the 6-min resting-state fMRI measurement, T2*-weighted echo-planar images (EPI) were recorded with a transversal orientation of 30° clockwise to the anterior commissures–posterior commissures (AC-PC)–line. For each subject, 240 images were acquired (repetition time [TR] = 1.5 s, echo time [TE] = 28 ms, flip angle = 80°, number of slices = 24, slice thickness = 4 mm, gap = 1 mm, voxel dimensions = 3 × 3 × 5 mm^3^, field of view (FOV) = 192 × 192 mm^2^, 64 × 64 in-plane resolution). We, additionally, acquired T1-weighted data (3D Magnetization Prepared Rapid Acquisition Gradient Echo [MPRAGE], sequence 192 sagittal slices, slice thickness = 1 mm, voxel dimensions = 1 × 1 × 1 mm^3^, FOV = 256 × 256 mm^2^, TR = 2300 ms, TE = 3.03 ms, TI = 900 ms, flip angle = 9°).

Structural and functional data were pre-processed and analyzed using the CONN-toolbox (Whitfield-Gabrieli and Nieto-Castanon 2012). The standard pre-processing pipeline includes the following: motion correction/realignment and unwarping (Andersson et al. 2001); slice-timing correction (Henson et al. 1999); outlier identification; unified segmentation and normalization (Ashburner and Friston 2005); and smoothing, using spatial convolution with a Gaussian kernel of 8 mm full width half maximum (FWHM). Within-subject differences in mean framewise displacement values were compared between sessions (for more details, please see Fig. S1 in the Supplementary Information). For denoising, the anatomical component-based noise correction method (aCompCor) was used, which included five noise components from cerebral white matter and cerebrospinal areas (Chai et al. 2012), 12 estimated subject-motion parameters (Friston et al. 1996), and scrubbing (Power et al. 2014) as well as constant and first-order linear session effects (Whitfield-Gabrieli and Nieto-Castanon 2012). Temporal frequencies below 0.01 Hz or above 0.09 Hz were removed from the BOLD signal (Hallquist et al. 2013).

**Fig. 2:**
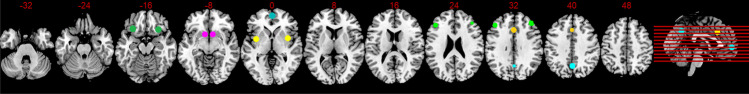
Schematic display of examined neural systems with corresponding nodes. Impulsive System (violet): Nucleus accumbens left (− 9.5, 12, − 7), Nucleus accumbens right (9, 12, − 7); Reflective System (green): lateral prefrontal cortex left (− 43, 33, 28), lateral prefrontal cortex right (41, 38 30), orbitofrontal cortex left (− 30, 24, − 17), orbitofrontal cortex right (29, 23, − 16); Salience Network (yellow): anterior cingulate Cortex (0, 22, 35), insular cortex left (− 36, 1, 0), insular cortex right (37, 3, 0): Default Mode Network (blue): medial prefrontal cortex (1, 55, − 3); posterior cingulate cortex (1, − 61, 38)

First-level analysis of the rsFC data was performed by using SCA (e.g., Fox et al. 2005; Greicius et al. 2003). A priori atlas regions (CONN default atlas combines FSL Harvard–Oxford atlas for cortical and subcortical areas and AAL atlas for cerebellar parcellation) were defined as seed regions from which the reference time course is formed, and which are correlated with the time courses of all other voxels in the brain. Thus, an FC map was generated for each individual, taking into account both positive and negative correlations. The seeds consisted of spheres with a diameter of 10 mm and are already implemented in the CONN toolbox (Whitfield-Gabrieli and Nieto-Castanon 2012). Since most intrinsic networks are lateralized (Agcaoglu et al. 2015), each hemisphere was examined separately (with the exception of midline structures). A schematic representation of the examined seeds and their coordinates is shown in Fig. 2.

For the second-level analysis, General Linear Model (GLM) analyses were performed. Between-subjects contrast (group variable: nalmefene/placebo vs. placebo/nalmefene [1, 0; 0, 1]) and within-subjects contrast (nalmefene > placebo or nalmefene < placebo; [1, -1] or [-1, 1]) were defined, resulting in a two-way ANOVA with main treatment effect (F-test). To control for multiple statistical testing, only results surviving multiple whole-brain corrections using FDR (*p* < 0.05) are reported. A voxel-threshold *(p* < 0.01) in combination with cluster-threshold (*p* < 0.05) was used. Further analysis of the questionnaires as well as the recording of side effects was conducted using the Statistical Package for the Social Sciences (SPSS) version 24.0 for Windows.

## Results

**Fig. 3:**
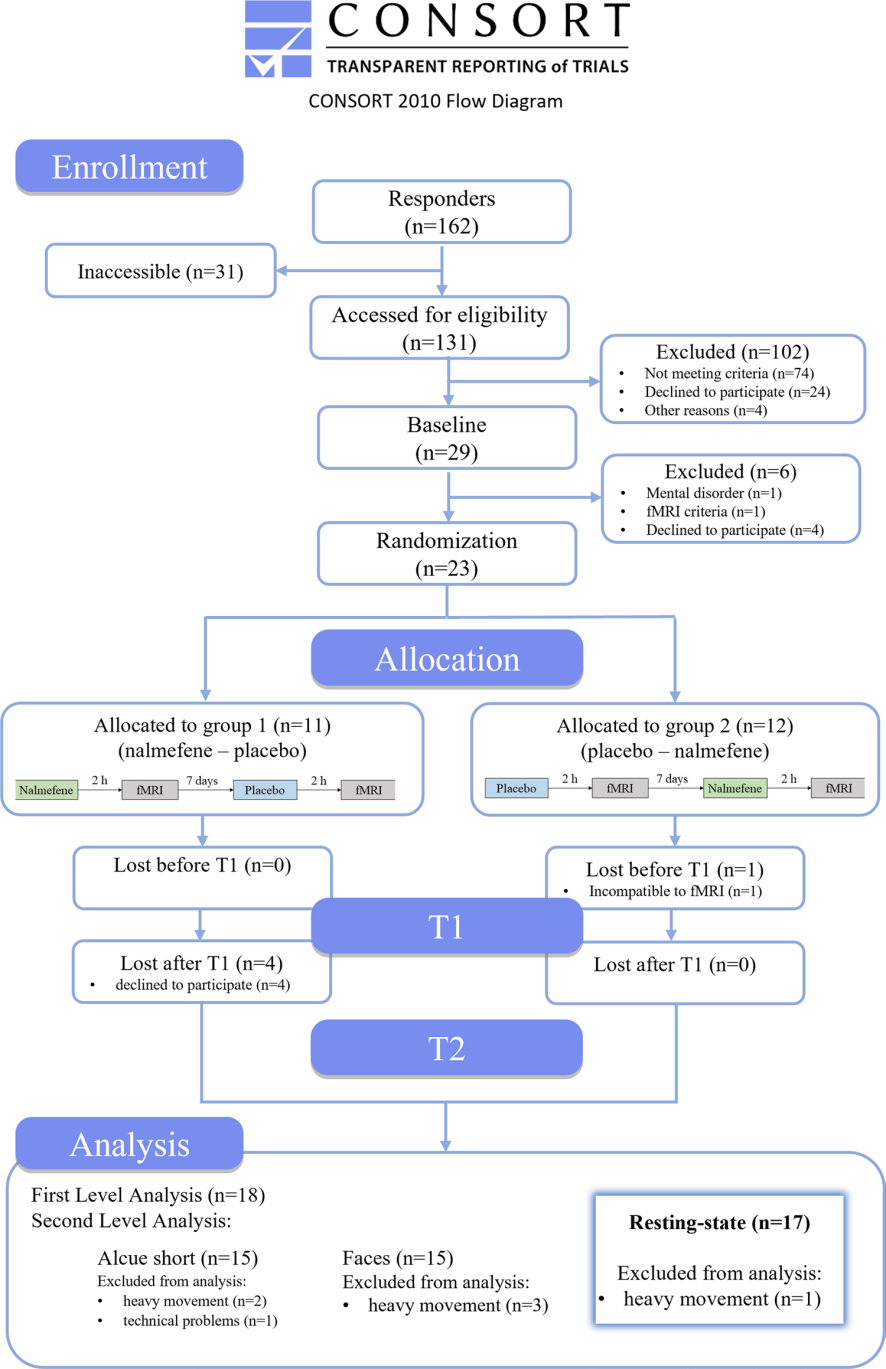
Enrollment process

From 131 screened persons, 23 were eligible to participate. Participants were randomized either to group (1) nalmefene–placebo (*n* = 11) or group (2) placebo–nalmefene (*n* = 12). A total of 18 participants successfully completed both sessions; however, one person could not be included in the analyses due to poor data quality. The enrollment process and dropouts are displayed in the CONSORT flow diagram (Fig. 3). The final study sample consisted of 17 participants, 65% male (*n* = 11) aged from 19 to 66 years (M = 51.3, SD = 13.7). For a comprehensive list of sample characteristics, please see Table 1.

**Table 1:** Sample characteristics

	*N*	*(%)*	*Min*	*Max*	*M*	*SD*
Age	17		19	66	51.29	13.74
Sex						
Male	11	(64.7)				
Female	6	(35.3)				
Smokers	9	(52.9)				
Alcohol consumption^1^						
Drinks / day^2^	17		0.71	15.12	6.49	4.44
Pure alcohol (g) / day	17		8.49	181.43	77.92	53.30
Drinking days	16		16	90	69.63	23.03
Drinks / day on drinking days^2^	16		1.48	15.29	8.24	4.41
Pure alcohol (g) / day on drinking days	16		17.77	183.50	102.47	52.98
Number of fulfilled DSM-5 criteria	15		4	8	6.33	1.35
ADS	17		1	15	8.65	4.44
AUDIT	17		10	28	17.24	5.78
FTND (only smokers)	9				3.44	3.01

^1^*A*lcohol consumption within the last 90 days collected by form 90 interview (Scheurich et al. 2005); ^2^number of standard drinks (12 g alcohol). *ADS*, alcohol dependence scale (Skinner and Allen 1982); *AUDIT*, alcohol use disorders identification test (Bohn et al. 1995); *FTND*, Fagerström test for nicotine dependence (Heatherton et al. 1991).

### Tolerability of nalmefene

Of all 23 participants, a total of 10 persons (33%) reported side effects after taking the study medication. They experienced between two and 11 symptoms (M = 5.8, SD = 2.7), e.g., Insomnia (*n* = 5), vertigo (*n* = 5), or nausea (*n* = 4). For details, please see Table S2 in the Supplementary Information.

### Resting‐state functional connectivity

**Fig. 4:**
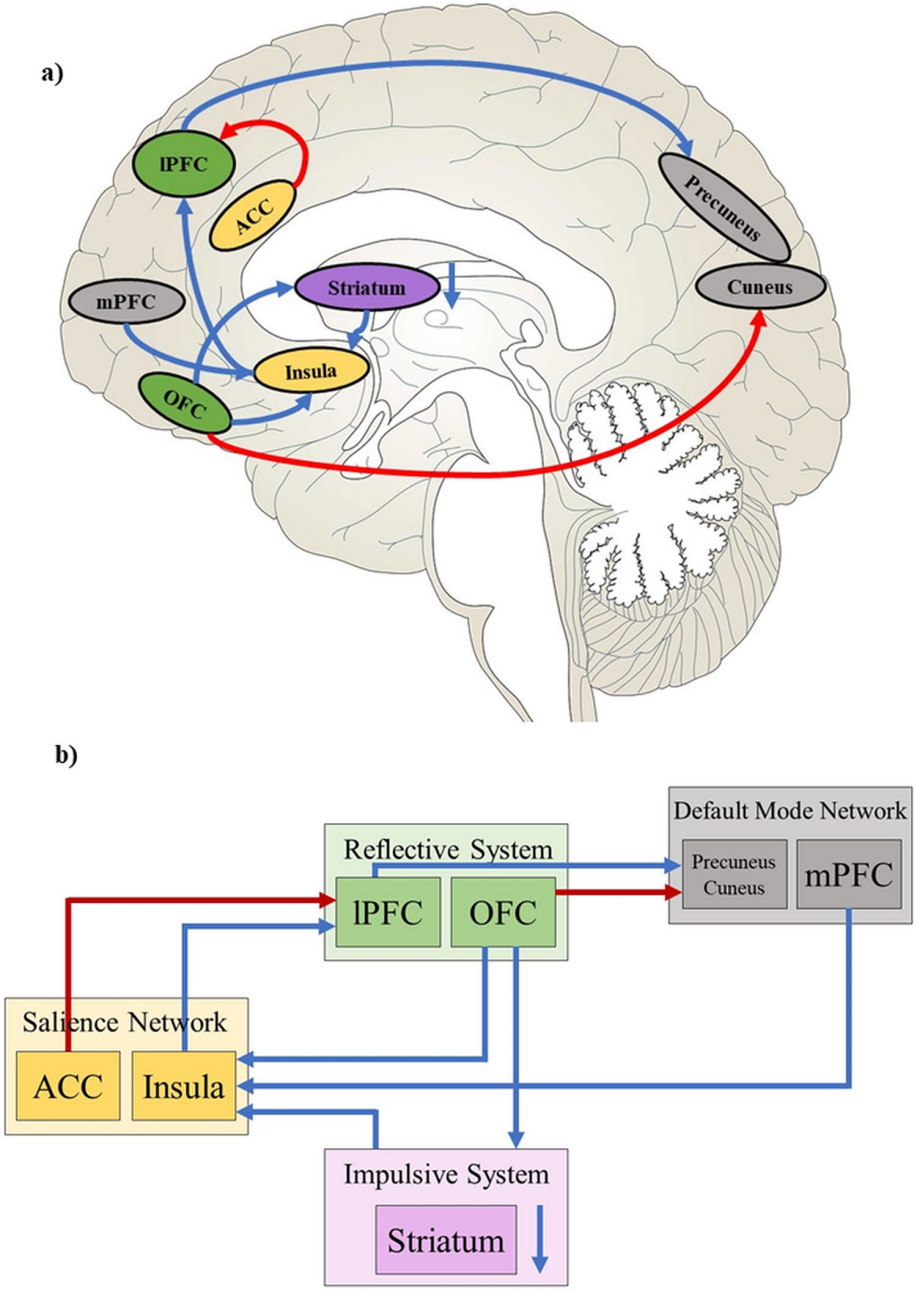
Effects of 18 mg nalmefene on the functional connectivity in key nodes of the neural systems. **a** Overview of the effects of nalmefene on resting-state functional connectivity (rsFC). Increased (red arrow) and decreased (blue arrow) functional connectivity between key nodes of the impulsive system (purple); reflective system (green); salience network (yellow); default mode network (gray). **b** Schematic representation explained from left to right: Increased rsFC between elements of the reflective system and ACC, which enhances inhibitory control. The ACC is responsible for conflict monitoring and error detection and reports to the dlPFC how much cognitive control is required. Reduced rsFC between the left insula and parts of the reflective system (insula–dlPFC; OFC–insula). This may prevent the embodied drug states represented in the insula from overpowering and hijacking the cognitive control system. Reduced coupling between the mPFC and the insula-IFG-network to strengthen inhibitory control. Reduced rsFC between right Nacc and the insula to reduce craving and may prevent the representation of interoceptive drug body states from affecting the Nacc, which could lead to drug-seeking behavior. Reduced rsFC within the striatum. By downregulating the impulsive system and possibly thereby normalizing the hypersensitized reward system, attentional bias and craving may be reduced. Decreased rsFC between OFC and Nacc to prevent the OFC from influencing the Nacc by presenting a high drug value or an incentive representation (wanting), which in turn can lead to a salience value of alcohol-related stimuli and promote approaching behavior. Increased rsFC between OFC and precuneus, which strengthens positive emotionality. Decreased rsFC between OFC and IFG, whose coupling is associated with anxiety. Reduced rsFC between right lPFC and precuneus, whose extended and excessive connectivity in AUD is associated with lack of inhibitory control and craving

**Fig. 5:**
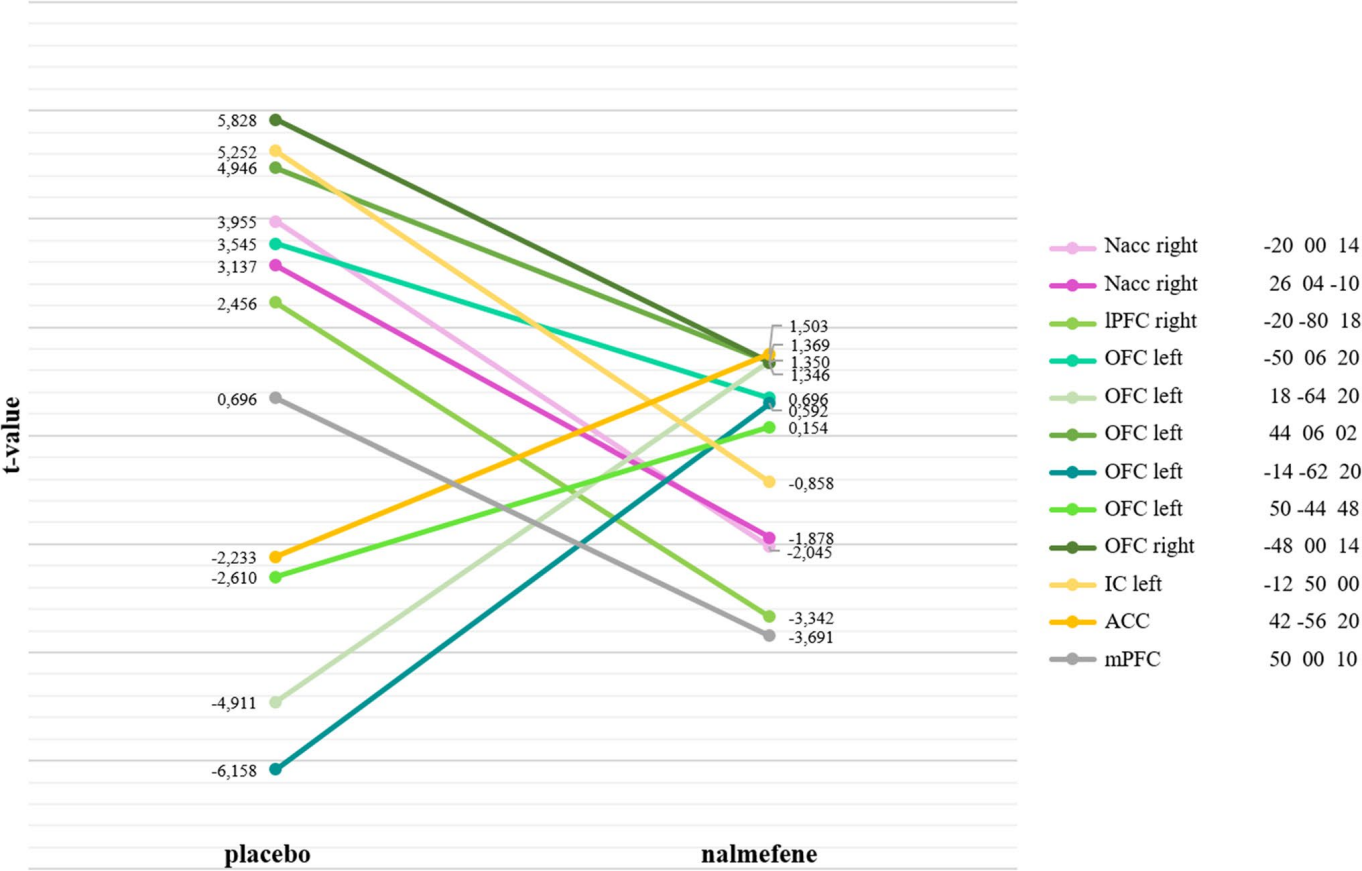
Changes in resting-state functional connectivity between placebo and nalmefene. *t*-values from the significant clusters (local maximum) reported in the results (FC placebo vs. nalmefene). MNI, Montreal Neurological Institute

For a schematic overview of the effects of nalmefene on the rsFC in key nodes of the neural systems, please refer to Fig. 4. The respective results relate to rsFC between the seed region and the rest of the brain after 18 mg nalmefene compared to placebo (contrast: nalmefene > placebo) and are reported for a voxel-wise-threshold of *p* < 0.01 combined with cluster-extent FDR-corrected *p*-value of *p* < 0.05. For the results that survive a more conservative voxel-wise-threshold of *p* < 0.001 or < 0.005, we refer to the Supplementary Information at the appropriate place. The rsFC brain maps for each condition separately are also included in the Supplemental Information (Fig. S2: nalmefene; Fig. S3: placebo). In addition, to show the change from placebo to nalmefene, rsFC was extracted from the significant clusters and presented in a line plot (please see Fig. 5).

#### The impulsive system

**Fig. 6:**
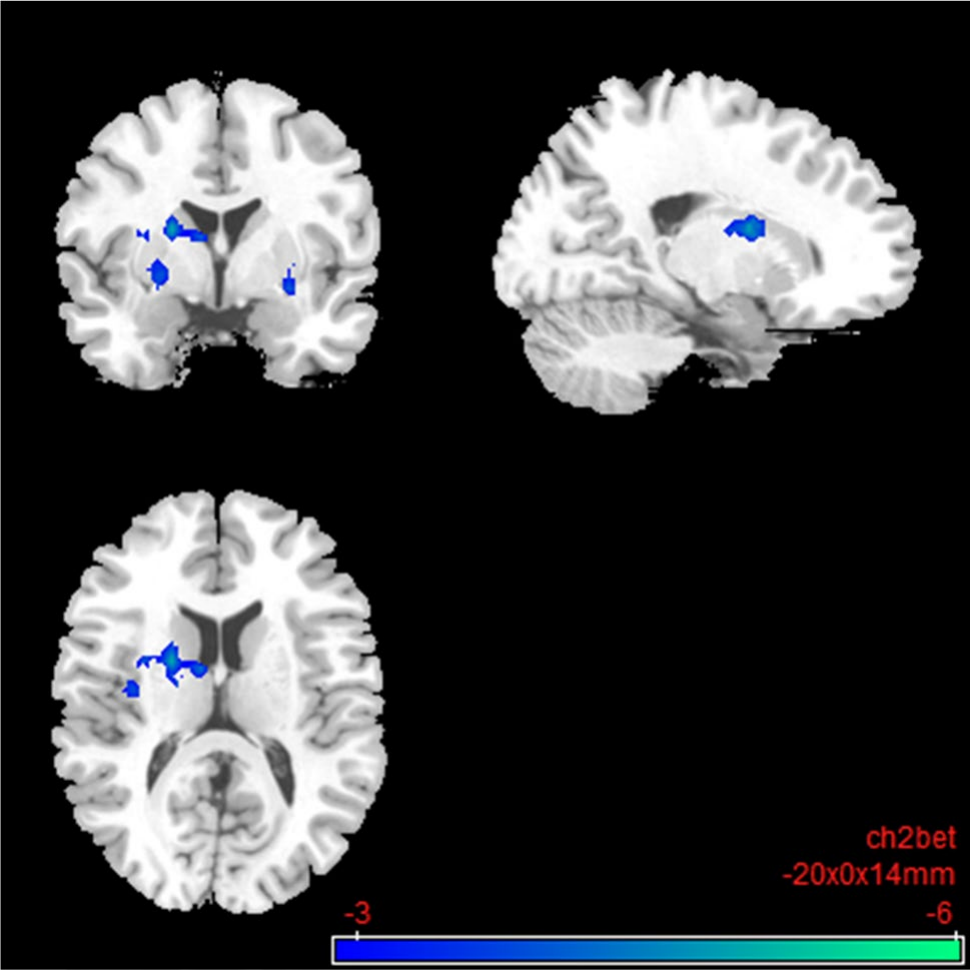
Impulsive system: brain regions with decreased resting-state functional connectivity between the seed region “right nucleus accumbens” and the rest of the brain after 18 mg nalmefene compared to placebo (contrast: nalmefene > placebo, MNI coordinates: − 20 00 14). Combined voxel-wise-threshold (*p* < .01) and cluster-extent threshold *k* > 460 Voxel, corresponding to *p*FDR < .05

Compared to the placebo condition, the rsFC in the nalmefene condition showed reduced FC within elements of the impulsive system. There was decreased connectivity between the right Nacc and putamen, pallidum, caudate, thalamus, amygdala, and insula (Table 2; Fig. 6). The results also survived a more conservative voxel-wise threshold of *p* < 0.005 (please see Table S3; Fig. S4 in the Supplementary Information). No significant results could be detected for the left Nacc.

**Table 2:** Impulsive system: resting-state functional connectivity with the seed region “right nucleus accumbens” after 18 mg nalmefene compared to placebo

Side	Brain areas	Size	MNI coordinates			t_max_	Cluster *p*FDR	Peak *p*-unc
			*x*	*y*	*z*			
L	Putamen, pallidum, caudate	578	− 20	00	14	− 5.22	0.0004	< 0.0001
L	Insula							
	Thalamus Claustrum							
R	Putamen, pallidum	460	26	04	− 10	− 5.39	0.0011	< 0.0001
R	Thalamus							
R	Amygdala							

Second level SCA results: resting-state functional connectivity between the seed region right Nucleus accumbens and the rest of the brain after 18 mg nalmefene compared to placebo (contrast: nalmefene > placebo). Combined voxel-wise-threshold (*p* < 0.01) and cluster-extent threshold *k* > 460 Voxel, corresponding to *p*FDR < 0.05; *MNI*, Montreal Neurological Institute.

#### The reflective system

**Fig. 7:**
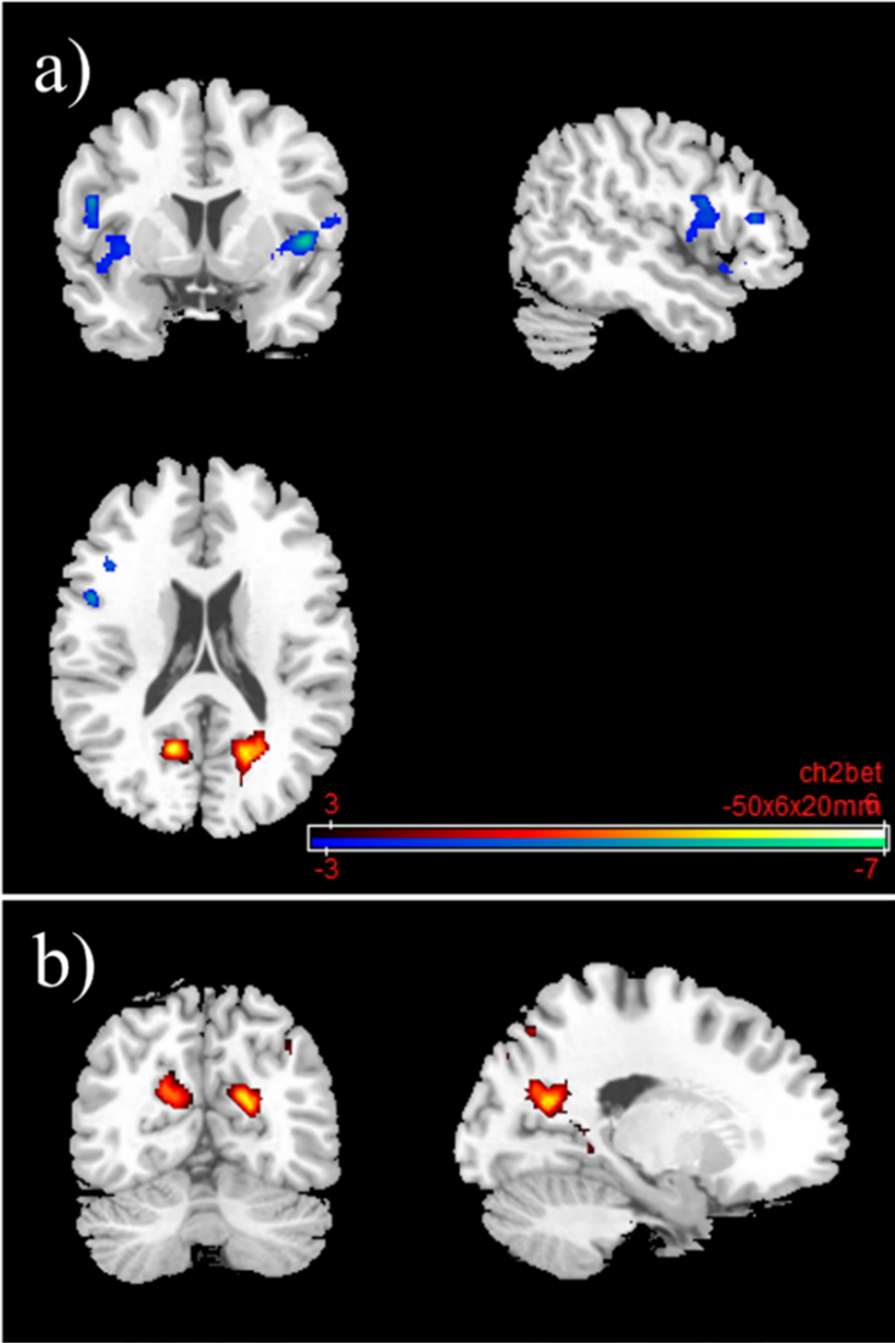
Reflective system: brain regions with decreased and increased resting-state functional connectivity between the seed region “left orbitofrontal cortex” and the rest of the brain after 18 mg nalmefene compared to placebo (contrast: nalmefene > placebo, MNI coordinates **a** − 50 06 20, MNI coordinates **b** 18 − 64 20). Combined voxel-wise-threshold (*p* < .01) and cluster-extent threshold *k* > 442 Voxel, corresponding to *p*FDR < .05

Under nalmefene, reduced rsFC between the right lPFC and a cluster consisting of the precuneus, and occipital regions was observed (Supplementary Information: Table S4; Fig. S5). No significant results could be detected for the left lPFC. The left OFC displayed reduced rsFC bilaterally to the insula and frontal regions (inferior frontal gyrus, precentral gyrus, and rolandic operculum) as well as the superior temporal gyrus. It also showed reduced rsFC to limbic regions, e.g., right putamen and pallidum. Increased connectivity was observed between the left OFC and two bilateral parietal/occipital clusters, consisting of posterior cingulate gyrus, precuneus, and cuneus. In addition, the left OFC showed increased connectivity to the right inferior and superior parietal lobule (Table 3; Fig. 7). For a more conservative voxel-wise threshold of *p* < 0.001, please see Table S5 and Fig. S6 in the Supplementary Information. The right OFC also showed a reduced FC to the insula and to frontal regions (inferior frontal gyrus, precentral gyrus, rolandic operculum) as well as to the postcentral gyrus (Supplementary Information: Table S6, Fig. S7).

**Table 3:** Reflective system: resting-state functional connectivity with the seed region “left orbitofrontal cortex” after 18 mg nalmefene compared to placebo

Side	Lobe	Brain areas	Size	MNI coordinates			t_max_	Cluster *p*FDR	Peak *p-*unc
				*x*	*y*	*z*			
L		Insula	1208	− 50	06	20	− 5.84	< 0.0001	< 0.0001
L	Frontal	Inferior frontal gyrus, precentral gyrus, rolandic operculum							
L	Temporal	Superior temporal gyrus, middle temporal gyrus							
R		Posterior cingulate gyrus, parahippocampal gyrus	569	18	− 64	20	5.58	0.0005	< 0.0001
R	Parietal	Precuneus							
	Occipital	Cuneus, lingual gyrus, calcarine							
R		Insula	548	44	06	02	− 6.59	0.0005	< 0.0001
R		Putamen, pallidum Claustrum							
R	Frontal	Inferior frontal gyrus, precentral gyrus, rolandic operculum							
	Temporal	Superior temporal gyrus							
		Posterior cingulate gyrus	503	− 14	− 62	20	5.67	0.0007	< 0.0001
L	Parietal	Precuneus, superior parietal lobule, inferior parietal lobule							
L	Occipital	Cuneus, superior occipital gyrus, middle occipital gyrus, calcarine							
R	Parietal	Precuneus, angular gyrus, inferior parietal lobule, superior parietal lobule, supramarginal gyrus	442	50	− 44	48	5.36	0.0015	< 0.0001
R	Occipital	Cuneus, superior occipital gyrus, middle occipital gyrus							

Second level SCA results: resting-state functional connectivity between the seed region left orbitofrontal cortex and the rest of the brain after 18 mg nalmefene compared to placebo (contrast: nalmefene > placebo). Combined voxel-wise-threshold (*p* < 0.01) and cluster-extent threshold *k* > 442 Voxel, corresponding to *p*FDR < 0.05; *MNI*, Montreal Neurological Institute.

#### The salience network

**Fig. 8:**
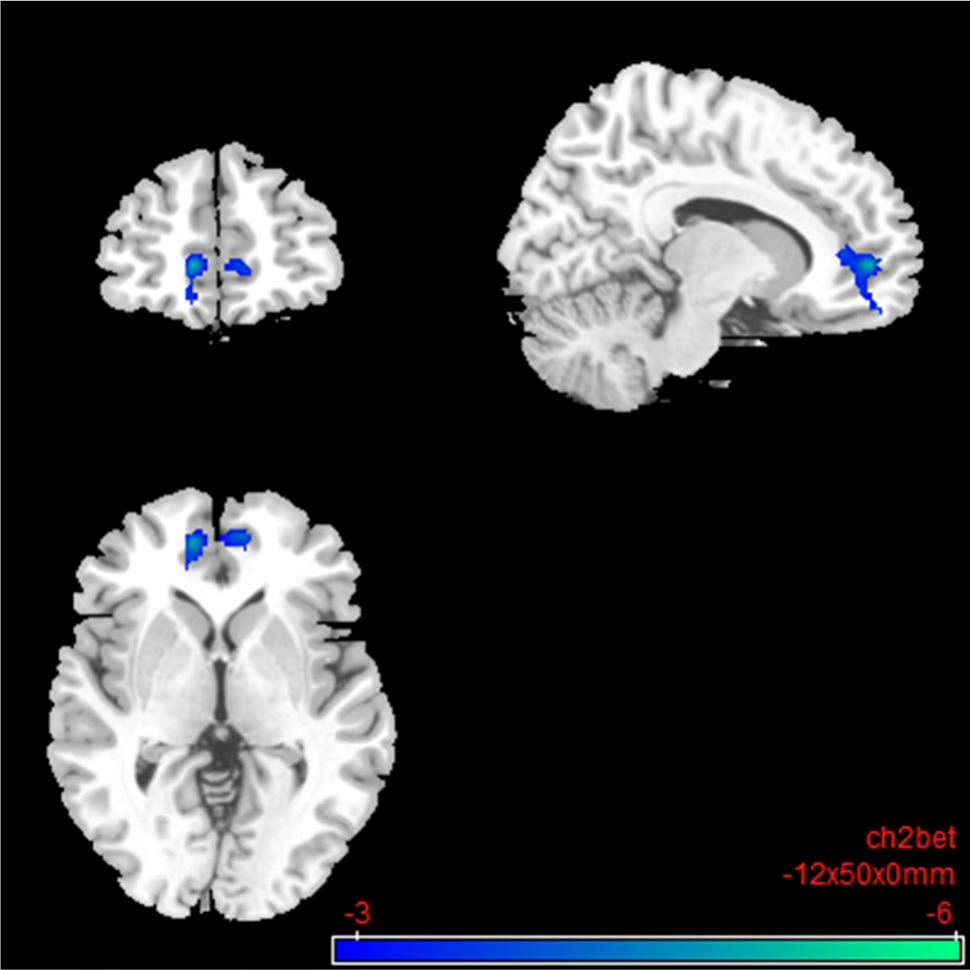
Salience network: brain regions with decreased resting-state functional connectivity between the seed region “left insular cortex” and the rest of the brain after 18 mg nalmefene compared to placebo (contrast: nalmefene > placebo, MNI coordinates: − 12 50 00). Combined voxel-wise-threshold *(p* < .01) and cluster-extent threshold *k* > 388 Voxel, corresponding to *p*FDR < .05

**Fig. 9:**
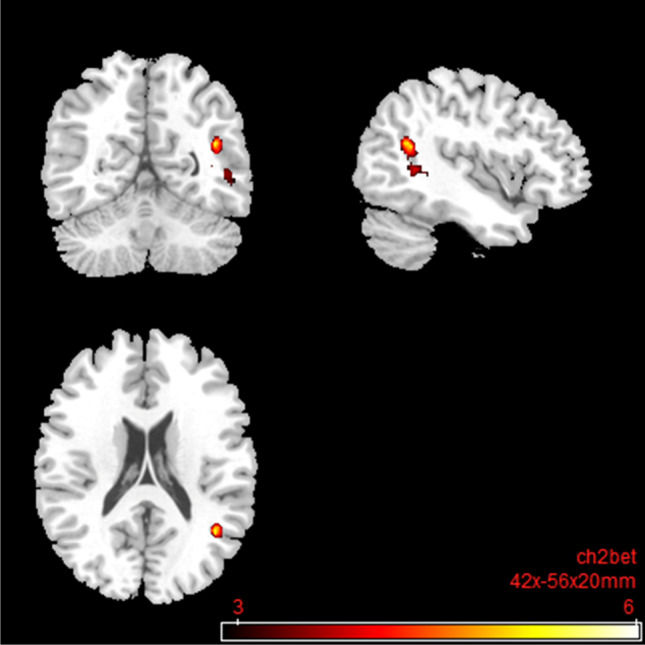
Salience network: brain regions with increased resting-state functional connectivity between the seed region “anterior cingulate cortex” and the rest of the brain after 18 mg nalmefene compared to placebo (contrast nalmefene > placebo, MNI coordinates: 42 − 56 20). Combined voxel-wise-threshold (*p* < .01) and cluster-extent threshold *k* > 372 Voxel, corresponding to *p*FDR < .05

Under nalmefene, the insular cortex showed reduced connectivity to a cluster of frontal (medial frontal and superior frontal gyrus) and paralimbic brain regions (anterior cingulate gyrus and dorsal ACC; Table 4; Fig. 8). Under nalmefene, the ACC showed an elevated rsFC to the right angular gyrus as well as middle temporal gyrus, superior temporal gyrus, inferior temporal gyrus, and parahippocampal gyrus (Table 5; Fig. 9).

**Table 4:** Salience network: resting-state functional connectivity with the seed region “left insular cortex” after 18 mg nalmefene compared to placebo

Side	Lobe	Brain areas	Size	MNI coordinates			t_max_	Cluster *p*FDR	Peak *p-*unc
				*x*	*y*	*z*			
L		Anterior cingulate, dorsal anterior cingulate cortex	388	− 12	50	00	− 5.22	0.0198	< 0.0001
L|R	Frontal	Medial frontal gyrus, superior frontal gyrus, anterior prefrontal cortex							

Second level SCA results: resting-state functional connectivity between the seed region left insular cortex and the rest of the brain after 18 mg nalmefene compared to placebo (contrast: nalmefene > placebo). Combined voxel-wise-threshold *(p* < 0.01) and cluster-extent threshold *k* > 388 Voxel, corresponding to *p*FDR < 0.05; *MNI*, Montreal Neurological Institute.

**Table 5:** Salience network: resting-state functional connectivity with the seed region “anterior cingulate cortex” after 18 mg nalmefene compared to placebo

Side	Lobe	Brain areas	Size	MNI coordinates			t_max_	Cluster *p*FDR	Peak *p-*unc
				*x*	*y*	*z*			
R	Parietal	Angular gyrus	372	42	− 56	20	5.76	0.0205	< 0.0001
R	Temporal	Middle temporal gyrus, superior temporal gyrus, inferior temporal gyrus, parahippocampal gyrus (9 V)							

Second level SCA results: resting-state functional connectivity between the seed region anterior cingulate cortex and the rest of the brain after 18 mg nalmefene compared to placebo (contrast: nalmefene > placebo). Combined voxel-wise-threshold (*p* < 0.01) and cluster-extent threshold *k* > 372 Voxel, corresponding to *p*FDR < 0.05; *MNI*, Montreal Neurological Institute.

#### The default mode network

**Fig. 10:**
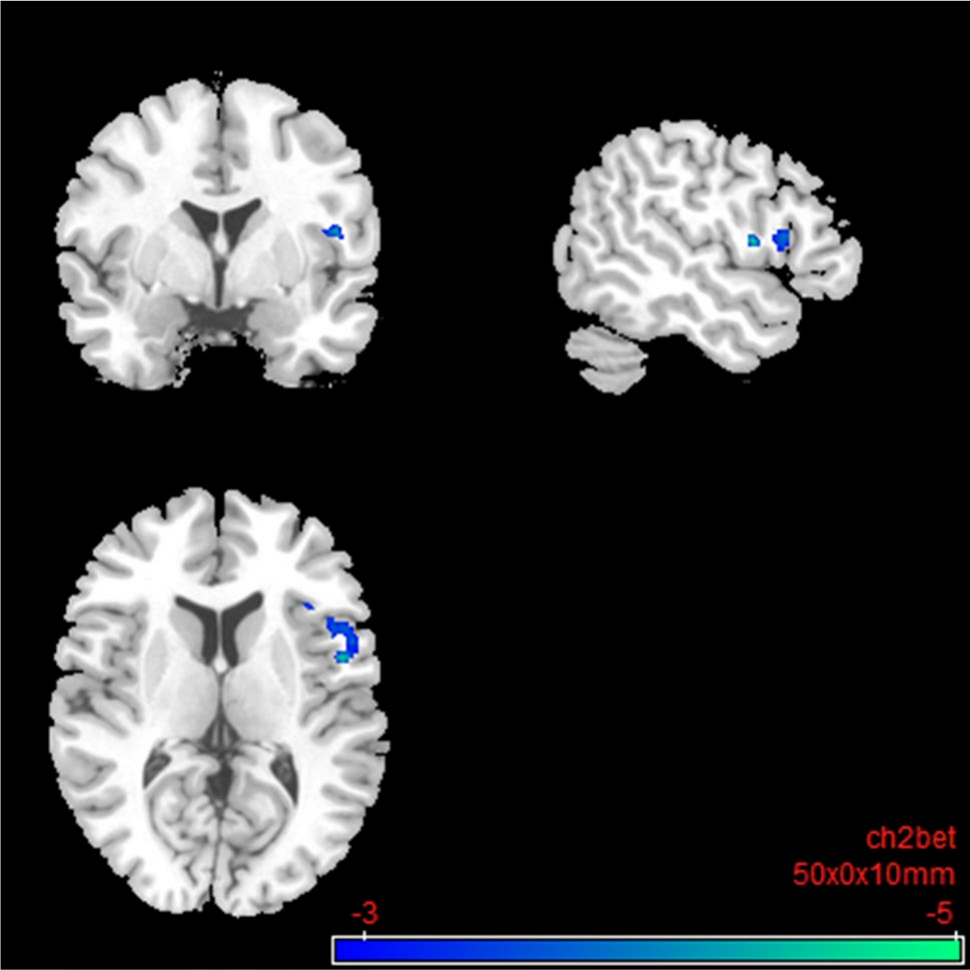
Default mode network: brain regions with decreased resting-state functional connectivity between the seed region “medial prefrontal cortex” and the rest of the brain after 18 mg nalmefene compared to placebo (contrast: nalmefene > placebo, MNI coordinates: 50 00 10). Combined voxel-wise-threshold (*p* < .01) and cluster-extent threshold *k* > 369 Voxel, corresponding to *p*FDR < .05

Under nalmefene, no significant changes in the rsFC between the PCC and the rest of the brain were found compared to the placebo. However, a significantly reduced FC between the mPFC and a frontal cluster consisting of the insula, inferior frontal gyrus, precentral gyrus, and rolandic operculum was observed (Table 6; Fig. 10). For a more conservative voxel-wise threshold of *p* < 0.001, please see Table S7, Fig. S8 in the Supplementary Information.

**Table 6:** Default mode network: resting-state functional connectivity with the seed region “medial prefrontal cortex” after 18 mg nalmefene compared to placebo

Side	Lobe	Brain areas	Size	MNI coordinates			t_max_	Cluster *p*FDR	Peak *p-*unc
				*x*	*y*	*z*			
R		Insula	369	50	00	10	− 4.82	0.0127	0.0002
R	Frontal	Inferior frontal gyrus (pars opercularis, triangularis, orbitalis), precentral gyrus, rolandic operculum							

Second level SCA results: resting-state functional connectivity between medial prefrontal cortex and the rest of the brain after 18 mg nalmefene compared to placebo (contrast: nalmefene > placebo). Combined voxel-wise-threshold (*p* < 0.01) and cluster-extent threshold *k* > 369 Voxel, corresponding to *p*FDR < 0.05; *MNI*, Montreal Neurological Institute.

## Discussion

The aim of this study was to investigate the effects of a single dose of nalmefene on rsFC in non-treatment seeking participants with AUD. The intrinsic rsFC is discussed as a potential biomarker for the understanding of addiction (Pariyadath et al. 2016; Wilcox et al. 2019). Moreover, rsFC could potentially be targeted with interventions, such as nalmefene, and thus contribute to the development of effective therapeutic treatments (Wilcox et al. 2019). In this study, key elements from rsFC networks were selected that are known to be compromised in AUD (Fox and Greicius 2010; Zhang and Volkow 2019; Zhu et al. 2017) and are also related to the triadic neurocognitive model of addiction (Noël et al. 2013): the impulsive system (Nacc), the reflective system (lPFC, OFC), and the salience system (insular cortex, ACC), as well as the DMN (mPFC, PCC). The present study was the first to show that a single dose of nalmefene appears to affect rsFC in components of these particular networks (see Fig. 4).

### The impulsive system

In AUD, behavior is controlled by drug-associated information, which can trigger automatic substance-related behavior through Pavlovian and instrumental learning mechanisms (Koob and Volkow 2010). These impulsive, fast, and ill-conceived reactions are thought to be related to a striatum-amygdala system (Noël et al. 2013; Zhu et al. 2017). In our study, nalmefene appears to reduce rsFC in components of this impulsive system and seems to down-regulate neural activity within the striatum. Our results show a significantly reduced FC between the right (but not the left) Nacc and the putamen, pallidum and caudate as well as the amygdala and insula. Interestingly, patients with AUD showed higher FC compared to healthy controls exactly within this impulsive amygdala striatum network (Zhu et al. 2017; Kohno et al. 2017). Increased FC within this impulsive system in AUD could represent the alcohol-induced increase in baseline sensitivity of the reward system. This sensitization could reflect an altered incentive salience for alcohol-associated stimuli (Sutherland et al. 2012) and, thereby, trigger craving and drug-seeking behavior. Interestingly, another part of the same study provided behavioral evidence that administration of nalmefene decreased alcohol craving. Thus, participants in the nalmefene condition showed both significantly lower subjective cue-induced craving (as measured by the Alcohol Urge Questionnaire) and attenuated reactivity to alcohol stimuli in the ventral striatum (Karl et al. 2021).

The insula plays an essential role in interoceptive processes and the representation of body states associated with drug effects (Naqvi et al. 2014). This representation of interoceptive drug body states is thought to reach the Nacc, where it affects the initiation of motivated and habitual action, which could lead to drug-seeking behavior (Naqvi and Bechara 2009). Cue-reactivity studies support this assumption, in which alcohol consumption is associated with greater activation in the striatum and insula (Myrick et al. 2004; Schacht et al. 2013). Our findings underpin the use of nalmefene to reduce alcohol consumption when confronted with alcohol-associated stimuli and are consistent with reward centered addiction models (Nestler 2005; Koob and Volkow 2010).

However, no significant results could be detected for the left Nacc. There is evidence that the right and left Nacc exhibit different rsFC patterns, with the right hemisphere being more connected to the insula, parahippocampal gyri, uncus, subcallosal regions, and cerebellum (Cauda et al. 2011). Other results indicate that left ventral striatum may be more involved in linking attentional responses to internally directed processes, whereas right ventral striatum contributes to attention directed to external behavioral contingencies, i.e., more sensitive to external conditioned cue stimuli (Zhang et al. 2017; Oberlin et al. 2016, 2015). The asymmetric lateralization of dopamine release in the Nacc is possibly caused by a functional regulatory role of the cerebellum. Thus, stimulation of cerebellar dentate nucleus was able to trigger dopamine release in the Nacc of the contralateral hemisphere, which was significantly greater in the right Nacc compared with the left (Holloway et al. 2019). High expression of μ-receptors and low expression of δ- and κ-opioid receptors were found in the human cerebellum (Peng et al. 2012). Therefore, nalmefene could block opioid receptors in the cerebellum; indirectly affect dopamine transmission in the right Nacc; and, thus, reduce cue-induced craving and approach behavior. Interestingly, nalmefene significantly attenuated not only BOLD responses in the striatum but also in the cerebellum during reward expectancy (Quelch et al. 2017), and naltrexone reduced activation in the right ventral striatum in response to alcohol-related stimuli (Schacht et al. 2017; Myrick et al. 2008). This also shows that the impulsive system is by no means limited to a striatum-amygdala-insula system. The extent to which other regions influence this circuit, and how nalmefene may affect them, requires further investigation. In addition, further investigation is needed to examine how the effects of nalmefene on rsFC driven by the relative involvement of different opioid receptors in the regulation of dopamine release and its influence on aversive and rewarding motivational aspects. For example, a recent study by Shokri-Kojori et al. (2021) showed that a naloxone-induced aversive and stressful state was associated with a dopamine increase in the dorsal, but not ventral, striatum. The effects of rsFC in the striatum and insula may also be influenced by direct physiological effects, as opioid receptor antagonists are known to affect not only dopamine release but also relative cerebral blood flow (CBF) for example in the insula and putamen (Shokri-Kojori et al. 2021). Therefore, future studies investigating the effects of nalmefene on rsFC could additionally combine this with dopamine and opioid receptor positron emission tomography (PET) and arterial spin labeling (ASL) or 15O-water PET for measuring CBF.

### The reflective system

While the impulsive system drives habitual and impulsive action, the reflective system should precisely control these impulses and suppress them if necessary (Noël et al. 2013). This ability to look beyond the immediate moment and incorporate long-term consequences in decision-making is a valuable skill, which individuals with SUD seem to lack. AUD patients are found to have significantly increased rsFC within the reflective system consisting of the left ECN and the OFCN compared with HC (Zhu et al. 2017). Interestingly, our study demonstrated significantly reduced rsFC between the right lPFC and the precuneus as well as occipital brain regions under nalmefene.

The precuneus represents a core region of the posterior DMN, which is seen to be involved in focusing attention on internal states (Leech and Sharp 2014), episodic memory, and conscious perception (Fletcher et al. 1995; Kjaer et al. 2001). In AUD, brain activity during the presentation of alcohol-related stimuli correlates with the degree of craving (Park et al. 2007) and the severity of dependence (Courtney et al. 2014). Alcohol stimuli may trigger visual memories of alcohol consumption, which are processed in the precuneus and may act as a conditioned stimulus (Park et al. 2007). Disturbed ECN–DMN connectivity could impair the ability to divert attention from internal rumination and craving (Zhang and Volkow 2019). In addition, higher anti-correlation between ECN and DMN lead to better treatment outcomes (Wilcox et al. 2019). The occipital cortex and cuneus have also been associated with cue-reactivity in addiction. Pathological gamblers showed increased activity in occipital regions (e.g., cuneus) but also in the dlPFC when exposed to sensory gambling cues. This has been associated with increased craving (Crockford et al. 2005). Individuals with AUD showed greater and extended dlPFC connectivity with the cuneus, which correlated with impairments in visuospatial working memory, possibly indicating a functional compensation mechanism due to alcohol-related impairments in the ECN and the visual network (Müller-Oehring et al. 2015). Based on this assumption, individuals with AUD may be unable to muster the necessary resources needed for cognitive control because of their inefficient strategies (Wang et al. 2018).

No significant results could be detected for the left hemisphere. According to a comprehensive study by Agcaoglu et al. (2015), the frontal networks are the most lateralized, with two right (inferior frontal and middle frontal gyrus) and two left components (inferior frontal gyrus). The seed of the network implemented in the CONN-Toolbox encompasses the coordinates of the middle frontal gyrus, which could explain the right lateralization. Other studies also show that dlPFC seems to be right lateralized (Nielsen et al. 2013). Interestingly, cue-induced alcohol craving appears to consistently activate areas of the left hemisphere, with the greatest asymmetry involving the left dlPFC. Evidence suggests, that a well-functioning right hemisphere is a protective factor against drug seeking behavior (Gordon 2016).

In addition, we observed reduced rsFC of the OFC between subcortical regions (e.g., striatum and insula); frontal brain regions (e.g., the inferior frontal gyrus); and parietal regions. In AUD, increased rsFC was observed within the OFCN and between the OFCN and the impulsive amygdala-striatum network, the latter did not survive correction for multiple comparisons (Zhu et al. 2017). This is consistent with findings in heroin addicts showing increased rsFC both within the medial OFC and between medial OFC and Nacc (Ma et al. 2010). The OFC encodes sensory stimuli by assigning them a certain value, both pleasant and unpleasant (Kringelbach 2005; Berridge and Kringelbach 2013). This modulation of reward values occurs possibly by regulating dopamine release in the striatum (Volkow et al. 2007; Wallis 2007). Our results indicate that nalmefene might normalize increased rsFC between the OFC and striatum, which could attenuate the higher salience level of alcohol-associated stimuli in individuals with AUD, reduce craving, and prevent approach behavior.

Furthermore, our results indicate that nalmefene may reduce rsFC between OFC and insula. Compared to social drinkers, individuals with AUD showed increased FC between the insula and medial OFC, possibly leading to impaired behavioral decision-making (Halcomb et al. 2019). Alcohol-related environmental stimuli could reactivate representations of the interoceptive effects of alcohol consumption in the insula (Naqvi and Bechara 2009). This “drug memory” could influence and override the OFC in its decision-making. It has been shown that the altered OFC activity in AUD leads to the inability to opt for long-term positive results (Boettiger et al. 2009). Nalmefene could reduce rsFC between OFC and insula and, thus, influence dysfunctional decision-making. This normalizing effect has already been demonstrated for naltrexone (Boettiger et al. 2009).

Moreover, our results show reduced rsFC between OFC and inferior frontal gyrus under nalmefene. Anxiety states caused by environmental stressors have been associated with a link between OFC, IFG, and amygdala; which in turn have been related to impaired goal-directed behavior in addiction (Gold et al. 2015; Ieong and Yuan 2017). By reducing FC between these brain regions, nalmefene may prevent anxiety states from affecting decision-making and goal-directed behavior.

Under nalmefene, our results also show increased rsFC between left OFC and parietal brain regions, which are attributed to the DMN (Andrews-Hanna et al. 2014). Interestingly, the personality trait “positive emotionality” is related to OFC activity and the corresponding cortical regions of the DMN (Volkow et al. 2011). In the context of SUD, positive emotionality is seen as a protective factor (Wills et al. 2001) as it is associated with a tendency towards positive mood, motivated behavior, and high reward sensitivity (Volkow et al. 2011)—characteristics that are affected by long-term drug use (Koob and Volkow 2010). Alcohol consumption is associated with an up-regulation of the k-receptor-dynorphin system in striatum, OFC, and dlPFC (Lindholm et al. 2007; Walker et al. 2012), which is mainly associated with dysphoria (Ciccocioppo 2002). This could lead to a depreciation of positive and hedonic states and an increase in negative affective states, impairing cognitive control and decision-making (Sirohi et al. 2012). Nalmefene seems to counteract this negative effect by binding to the k-receptors (Quelch et al. 2017). It is possible that nalmefene may, thus, increase rsFC in regions that contribute to positive emotionality.

### The salience network

Regarding the SN, both reduced rsFC (insula) and increased (ACC) rsFC were found under nalmefene. The left insula showed reduced rsFC to frontal regions, which are part of the reflective system, as well as to paralimbic regions. Interestingly, an increased rsFC between the SN, the ECN, and the amygdala-striatum-network has been demonstrated in AUD compared to HC (Zhu et al. 2017). The insula seems to contribute to the maintenance of addiction by translating interoceptive signals into a subjective and conscious feeling that individuals experience as craving. This highly embodied experience is thought to overpower the cognitive control system and may lead to drug-seeking or approach behavior (Naqvi and Bechara 2009). The insula could, thus, sensitize the activity of the impulsive system and simultaneously undermine the reflective system in its planning and decision-making processes. This is supported by the finding that impulsive decisions are associated with higher activity in the striatum, mPFC, and anterior insula (Lim et al. 2017). Nalmefene may interrupt the coupling of the insula to brain regions, which are part of the reflective system. This could prevent interoceptive signals from hijacking the cognitive resources that are important for decision-making or inhibitory control. There were no significant results for the right insula. Studies show that the attentional network is also highly lateralized. In some cases, a primary right lateralization of the SN was found (Nielsen et al. 2013), and in other cases, components that were strongly left or right lateralized (Agcaoglu et al. 2015). The left hemisphere appears to be associated with approach or appetitive behavior. Studies show that craving and “wanting” triggered by cue stimuli more often activate left frontal areas. Thus, both lower left hemisphere activation for craving and stronger impulse control in the right hemisphere could serve as protective factors against drug use (Gordon 2016).

In addition, increased rsFC between the ACC and brain regions related to the reflective system was shown under nalmefene. Interestingly, a study on patients with AUD and attention deficit hyperactivity disorder (ADHD) showed impaired connectivity between ACC and PFC, compared to HC, which was associated with higher severity of AUD or ADHD. This may indicate impaired inhibition—a common feature of both disorders—and that comorbidity could exacerbate this impairment (Farré-Colomés et al. 2021). The ACC is thought to be involved in inhibitory control (Ma et al. 2010) via conflict monitoring (Ridderinkhof et al. 2004) and error detection (Carter et al. 1998). Indeed, top-down cognitive control appears to occur mainly in the dlPFC; however, the ACC is also crucial, as it communicates the degree of cognitive control currently required (Ma et al. 2010). Therefore, higher cognitive control could result from closer coupling. Nalmefene could, thus, weaken one part of the salience system (insula) and strengthen the other part (ACC), which could partially normalize the dynamic interaction between the impulsive and reflective systems.

### The default mode network

Deficiencies, both within the DMN and interaction with other networks, appear to cause affective and cognitive impairments that promote craving and relapse in SUD (Zhang and Volkow 2019). Contrary to our expectations, we could not detect any significant changes between the PCC and other regions of the brain. Regarding the anterior DMN, reduced rsFC between the mPFC and frontal regions, such as the insula and inferior frontal gyrus, was displayed under nalmefene.

In patients with AUD, a trend towards increased rsFC between the anterior DMN and the left ECN was observed compared to HC (Zhu et al. 2017). Disturbed DMN suppression and impaired ECN–DMN connectivity is associated with poor cognitive functioning. Thus, the strong involvement of the DMN during withdrawal appears to prevent the cognitive control mediated by the ECN (Zhang and Volkow 2019). The insula, areas of the lPFC, and parietal regions form a unified task-activation ensemble (Seeley et al. 2007), which is often co-activated during cognitive tasks of attention and response selection (Menon et al. 2001). Reduced coupling between mPFC and the insula–IFG network could restore the anti-correlation of the two networks. For example, naltrexone has also been shown to reduce FC between the ECN and DMN, which has been associated with reduced drug use (Kohno et al. 2019). Furthermore, studies identified a neural circuit, consisting of mPFC, insula, and Nacc, which mediates aversion-resistant alcohol seeking and compulsive alcohol consumption (Grodin et al. 2018; Seif et al. 2013). By reducing rsFC between the mPFC and insula, nalmefene may be able to enhance the cognitive resources that are important for inhibitory control of compulsive behavior.

## Limitations

The selection of seeds in the present study was defined a priori, based on the triadic neurocognitive model of addiction (Noël et al. 2013) and previous results (Zhu et al. 2017). However, the knowledge and information derived from the fcMap is limited to the selection of the seed region. SCA is, thus, susceptible to bias. To study FC patterns in the entire brain, model-free methods, such as ICA, are recommended (van den Heuvel and Hulshoff Pol 2010; Lv et al. 2018). In addition, the resting-state measurement was performed after two task-based experiments. The two tasks (cue-reactivity and emotional processing) could have impacted rsFC fMRI. Although this is a placebo-controlled study, a complete blinding must be questioned in view of the reported side effects. In order to achieve reliable blinding, an “active placebo” would be useful, which would only induce the corresponding side effects. However, due to ethical considerations, active placebos are rarely used. Moreover, our within-subject design does not allow to assess whether there is indeed a normalization of FC patterns by nalmefene. Based on the results of other studies, the change in FC seems to be in the right direction, but without a control group, “normalization” cannot be assessed. Our sample consisted of mostly males and individuals who smoke. Both might reduce generalizability of the findings (e.g., Li et al. 2012; Filippi et al. 2013; Weissman-Fogel et al. 2010; Vergara et al. 2017). Overall, the sample size is quite modest and has a wide range in age and drinking. It would be appropriate to replicate the study with a larger sample and more stringent thresholds, considering possible differences between male and female participants.

## Conclusion

Intrinsic rsFC has been proposed as a potential biomarker for understanding addiction; therefore, interest is increasingly shifting towards the identification and therapeutic modification of rsFC biomarkers. The present study indicates that nalmefene impacts RSN and could change the interaction between addiction-related neural systems in non-treatment seeking individuals with AUD. Nalmefene might normalize rsFC by weakening the impulsive system associated with ill-conceived impulsive behaviors and habits. Simultaneously, it might strengthen the reflective system, which is responsible for cognitive control. In this way, nalmefene could reduce craving, as well as automatic-approach behavior, and at the same time restore control over alcohol consumption. This could contribute to an effective reduction in drinking. Overall, the reduction of alcohol consumption using nalmefene is still in its infancy and further research is required to expand the range of treatments for AUD. Nevertheless, this study’s results provide the neurobiological basis of the mechanisms of action and effectiveness of nalmefene.

## Supplementary Information

Below is the link to the electronic supplementary material.

Supplementary file1 (DOCX 5.81 MB)
