# Supplementary material for: The effects of nalmefene on the impulsive and reflective system in alcohol use disorder: A resting-state fMRI study
Source: Psychopharmacology (Berl). 2022 Apr 15;239(8):2471–89. doi: 10.1007/s00213-022-06137-1 (PMC9293828; doi:10.1007/s00213-022-06137-1)
Supplement: Supplementary file 1 — Supplementary file1 (DOCX 5.81 MB) [file 213_2022_6137_MOESM1_ESM.docx]

# **Supplementary Information**

## **Table S1**

*Inclusion and Exclusion Criteria*

| Inclusion criteria | Exclusion criteria |
| --- | --- |
| - man or woman - age between 18 and 70 years - AUD according to DSM 5 and - heavy drinking: alcohol consumption ≥ 60g (men) and ≥ 40g (women) minimum of 5 days/week - right-handedness - normal or corrected to normal vision - signed written informed consent | - psychotropic medication within the last 14 days - severe withdrawal symptoms (CIWA-Ar >4) - intoxication (breath alcohol > 0.3 ‰) - common exclusion criteria for MRI (e.g. metal, claustrophobia) - positive drug screening (opioids, cannabinoids, benzodiazepines, barbiturates, cocaine, amphetamines) - pregnancy - contraindications for nalmefene - previous severe withdrawal/withdrawal complications - previous inpatient detoxification treatment - Axis I psychiatric diagnoses (other than AUD/TUD in the last 12 months) - neurological disorders, brain injury |

*Note*. CIWA-Ar = Clinical Institute Withdrawal Assessment of Alcohol Scale, Revised (Sullivan et al. 1989)

**Fig. S1**

*Within-Subject Differences of Framewise Displacement between Sessions*


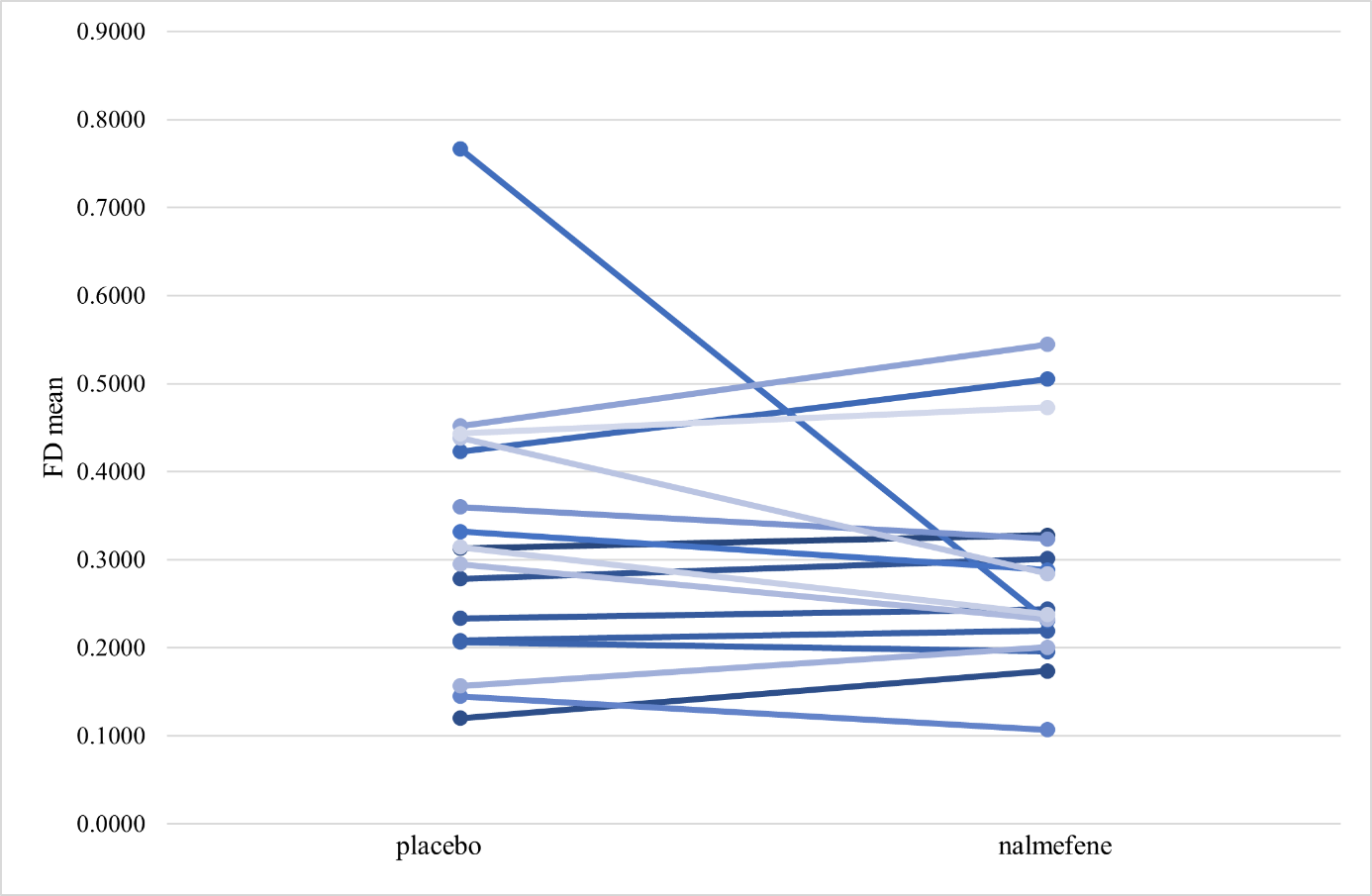


*Note*. Differences in mean framewise displacement (FD) values of each subject between the session ‘placebo’ and ‘nalmefene’. Mean FD was slightly higher in the placebo condition (M=.323, *SD*=.156) than in the nalmefene condition (M=.288, *SD*=.119). However, a paired sample t-test showed that this difference was not statistically significant, t(16) = 1.012, p = .326, 95% confidence interval [-.0384, .1085].

## **Table S2**

## *Nalmefene Side Effects*

| Symptom | Number of participants | % of overall side effects | % of all participants |
| --- | --- | --- | --- |
| Insomnia | 5 | 50 | 21,7 |
| Vertigo | 5 | 50 | 21,7 |
| Nausea | 4 | 40 | 17,4 |
| Faintness/drowsiness/tiredness | 4 | 40 | 17,4 |
| Appetite loss | 3 | 30 | 13,0 |
| Headache | 2 | 20 | 8,7 |
| Tunnel vision | 2 | 20 | 8,7 |
| Tenseness | 2 | 20 | 8,7 |
| Restlessness | 2 | 20 | 8,7 |
| irritability/aggressiveness | 2 | 20 | 8,7 |
| perceptional disturbance | 2 | 20 | 8,7 |
| attention deficit/lack of concentration | 2 | 20 | 8,7 |
| depersonalisation | 2 | 20 | 8,7 |
| derealisation | 1 | 10 | 4,3 |
| skin tingle | 1 | 10 | 4,3 |
| numbness (of body parts) | 1 | 10 | 4,3 |
| body perceptional disturbances | 1 | 10 | 4,3 |
| arrest of thought | 1 | 10 | 4,3 |
| stimulus satiation | 1 | 10 | 4,3 |
| prolongated reaction time | 1 | 10 | 4,3 |
| panic/anxiety | 1 | 10 | 4,3 |
| diarhhea | 1 | 10 | 4,3 |
| dry throat | 1 | 10 | 4,3 |
| increased salivation | 1 | 10 | 4,3 |
| subsultus | 1 | 10 | 4,3 |
| palpitation | 1 | 10 | 4,3 |
| hot and cold feeling | 1 | 10 | 4,3 |
| cold sweat | 1 | 10 | 4,3 |
| hyperhidrosis | 1 | 10 | 4,3 |
| nervousness/uneasiness | 1 | 10 | 4,3 |
| impairment in daily life | 1 | 10 | 4,3 |

## **Fig. S2**

*Resting-State Functional Connectivity after 18mg Nalmefene*

## **
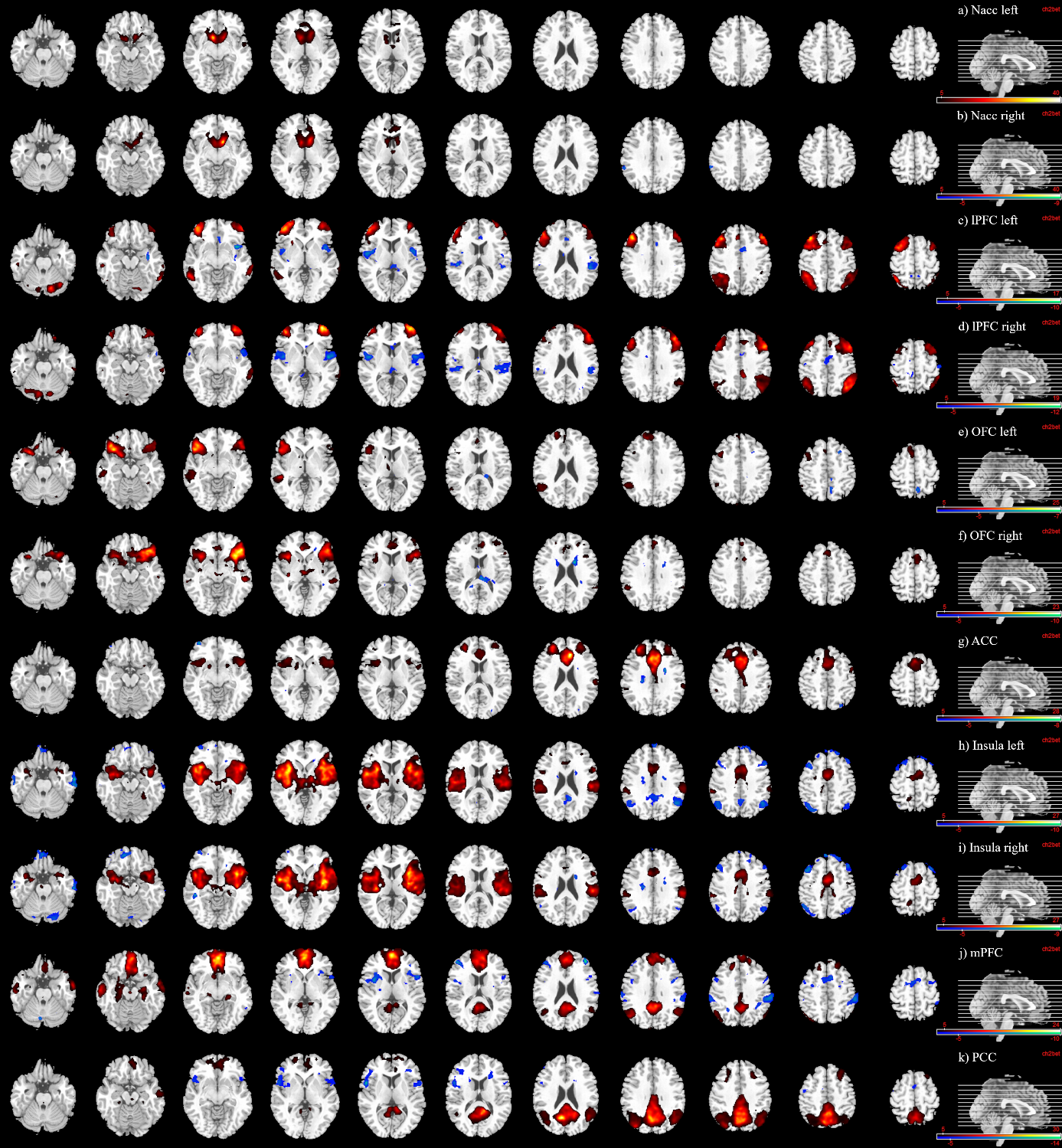
**

*Note.* a) Nucleus accumbens left b) Nucleus accumbens right c) lateral prefrontal cortex left d) lateral prefrontal cortex right e) orbitofrontal cortex left f) orbitofrontal cortex right g) anterior cingulate cortex h) insular cortex left i) insular cortex right j) medial prefrontal cortex k) posterior cingulate cortex

**Fig. S3**

*Resting-State Functional Connectivity after Placebo*


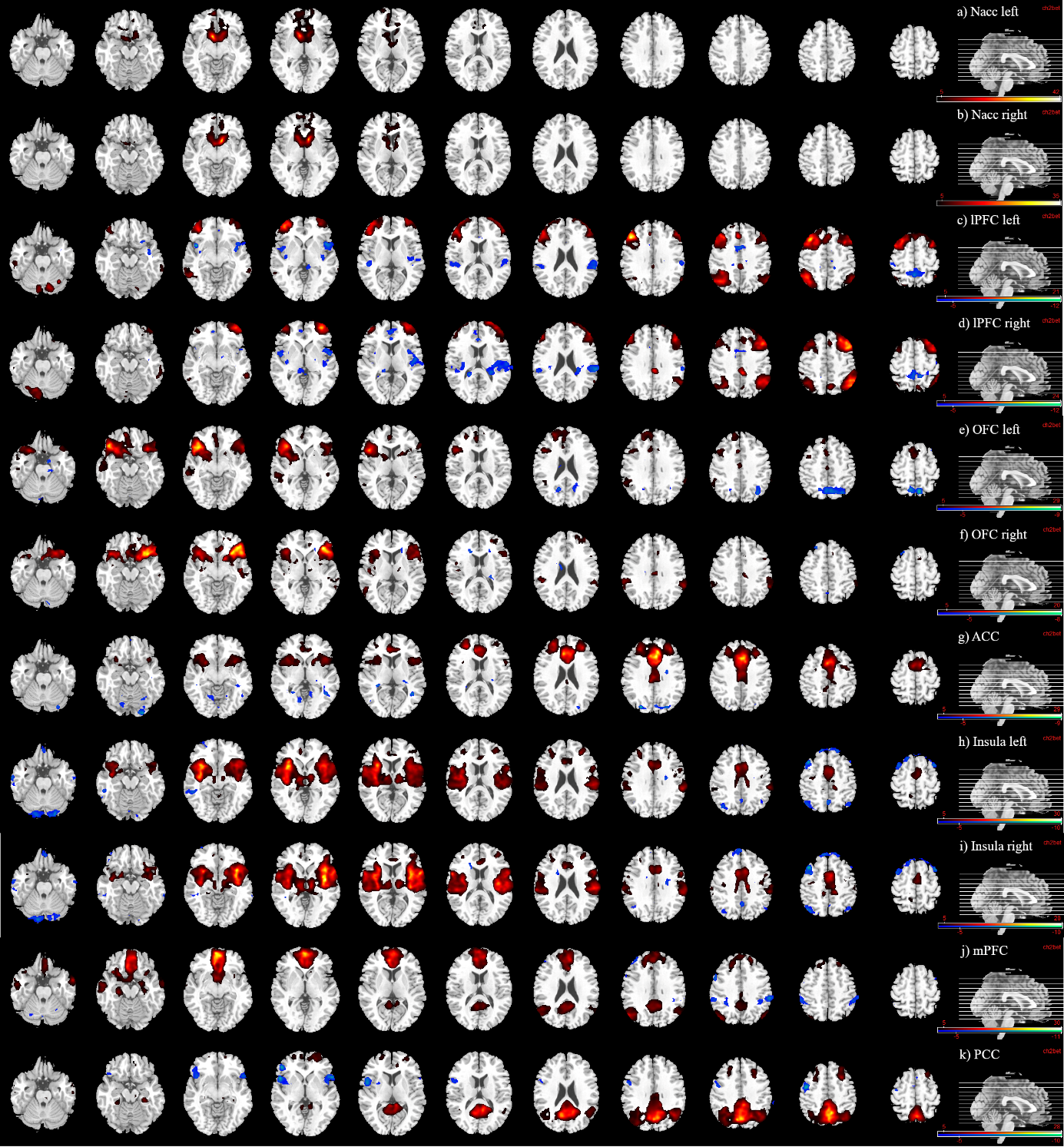


*Note.* a) Nucleus accumbens left b) Nucleus accumbens right c) lateral prefrontal cortex left d) lateral prefrontal cortex right e) orbitofrontal cortex left f) orbitofrontal cortex right g) anterior cingulate cortex h) insular cortex left i) insular cortex right j) medial prefrontal cortex k) posterior cingulate cortex

## **Table S3**

*Impulsive System: Resting-State Functional Connectivity with the Seed Region “Right Nucleus accumbens” after 18mg Nalmefene compared to Placebo*

| Side | Lobe | Brain Areas | Size | MNI Coordinates | | | t_max_ | Cluster  *p*FDR | Peak  *p*-unc |
| --- | --- | --- | --- | --- | --- | --- | --- | --- | --- |
|  |  |  |  | *x* | *y* | *z* |  |  |  |
| R |  | Putamen, Pallidum, Thalamus, Amygdala | 277 | 26 | 04 | -10 |  | .0045 | < .0001 |
| L |  | Putamen, Caudate, Pallidum, Insula | 253 | -20 | 00 | 14 |  | .0045 | < .0001 |

*Note*: Second level SCA results: resting-state functional connectivity between the seed region right Nucleus accumbens and the rest of the brain after 18mg nalmefene compared to placebo (contrast: nalmefene > placebo). Combined voxel-wise-threshold (*p* < .005) and cluster-extent threshold *k* > 25 Voxel, corresponding to *p*FDR < .05; MNI = Montreal Neurological Institute.

## **Fig. S4**

*Impulsive System: Resting-State Functional Connectivity with the Seed Region “Right Nucleus accumbens” after 18mg Nalmefene Compared to Placebo*

*
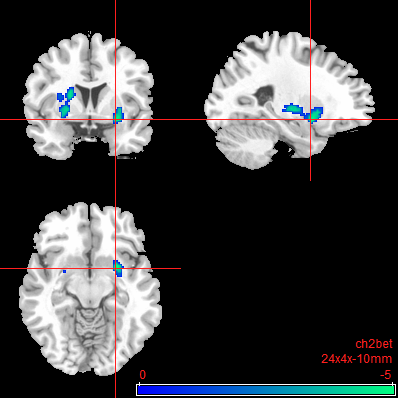
*

*Note.* Brain regions with decreased resting-state functional connectivity between the seed region right Nucleus accumbens and the rest of the brain after 18mg nalmefene compared to placebo (contrast: nalmefene > placebo, MNI coordinates: 26 04 -10). Combined voxel-wise-threshold (*p* < .005) and cluster-extent threshold *k* > 25 Voxel, corresponding to *p*FDR < .05

## **Table S4**

*Reflective System: Resting-State Functional Connectivity with the Seed Region “right Lateral Prefrontal Cortex” after 18mg Nalmefene Compared to Placebo*

| Side | Lobe | Brain Areas | Size | MNI Coordinates | | | t_max_ | Cluster  *p*FDR | Peak  *p*-unc |
| --- | --- | --- | --- | --- | --- | --- | --- | --- | --- |
|  |  |  |  | *x* | *y* | *z* |  |  |  |
| L | Parietal | Precuneus | 318 | -20 | -80 | 18 | -4.66 | .0329 | .0003 |
| L | Occipital | Superior occipital gyrus, Middle occipital gyrus, Cuneus |  |  |  |  |  |  |  |

*Note*: Second level SCA results: resting-state functional connectivity between the seed region right lateral prefrontal cortex and the rest of the brain after 18mg nalmefene compared to placebo (contrast: nalmefene > placebo). Combined voxel-wise-threshold (*p* < .01) and cluster-extent threshold *k* > 318 Voxel, corresponding to *p*FDR < .05; MNI = Montreal Neurological Institute.

## **Fig. S5**

*Reflective System: Resting-State Functional Connectivity with the Seed Region “Right Lateral Prefrontal Cortex” after 18mg Nalmefene Compared to Placebo*


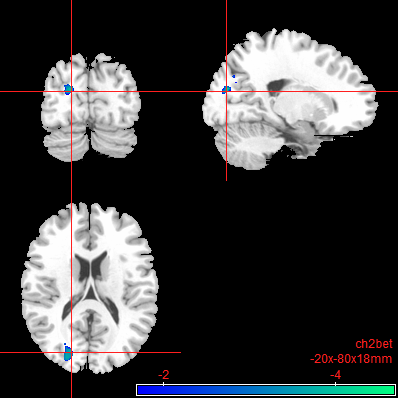


*Note.* Brain regions with decreased resting-state functional connectivity between the seed region right lateral prefrontal cortex and the rest of the brain after 18mg nalmefene compared to placebo (contrast: nalmefene > placebo, MNI coordinates: -20 -80 18). Combined voxel-wise-threshold (*p* < .01) and cluster-extent threshold *k* > 318 Voxel, corresponding to *p*FDR < .05

## **Table S5**

*Reflective System: Resting-State Functional Connectivity with the Seed Region “Left Orbitofrontal Cortex” after 18mg Nalmefene Compared to Placebo*

| Side | Lobe | Brain Areas | Size | MNI Coordinates | | | t_max_ | Cluster  *p*FDR | Peak  *p*-unc |
| --- | --- | --- | --- | --- | --- | --- | --- | --- | --- |
|  |  |  |  | *x* | *y* | *z* |  |  |  |
| R | Parietal | Precuneus | 155 | 18 | -64 | 20 |  | .0037 | < .0001 |
|  | Occipital | Cuneus, Calcarine |  |  |  |  |  |  |  |
| R |  | Insula, Central Opercular Cortex, Frontal Operculum | 112 | 44 | 06 | 02 |  | .0079 | < .0001 |
| L | Parietal  Occipital | Precuneus  Calcarine (1 Voxel) | 105 | -14 | -62 | 20 |  | .0079 | < .0001 |
| L | Frontal | Insula, Frontal Operculum, Inferior Frontal Gyrus | 105 | -38 | 12 | 02 |  | .0079 | .0002 |

*Note*: Second level SCA results: resting-state functional connectivity between the seed region left Orbitofrontal Cortex and the rest of the brain after 18mg nalmefene compared to placebo (contrast: nalmefene > placebo). Combined voxel-wise-threshold (*p* < .001) and cluster-extent threshold *k* > 105 Voxel, corresponding to *p*FDR < .05; MNI = Montreal Neurological Institute.

## **Fig. S6**

*Reflective System: Resting-State Functional Connectivity with the Seed Region “Left Orbitofrontal Cortex” after 18mg Nalmefene Compared to Placebo*


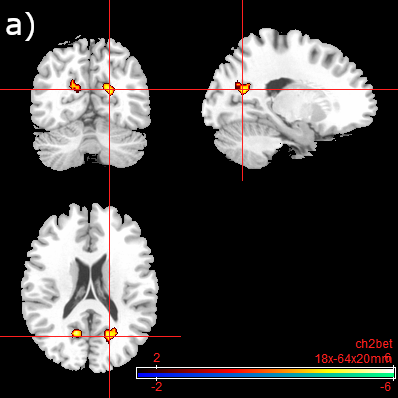

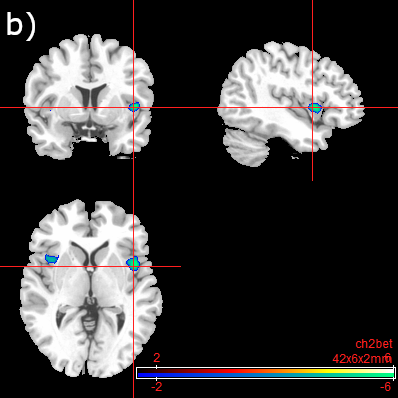


*Note.* Brain regions with increased and decreased resting-state functional connectivity between the seed region left orbitofrontal cortex and the rest of the brain after 18mg nalmefene compared to placebo (contrast: nalmefene > placebo, MNI coordinates: a) 18 -64 20; b) 44 06 02). Combined voxel-wise-threshold (*p* < .001) and cluster-extent threshold *k* > 105 Voxel, corresponding to *p*FDR < .05

## **Table S6**

*Reflective System: Resting-State Functional Connectivity with the Seed Region “Right Orbitofrontal Cortex” after 18mg Nalmefene Compared to Placebo*

| Side | Lobe | Brain Areas | Size | MNI Coordinates | | | t_max_ | Cluster  *p*FDR | Peak  *p-*unc |
| --- | --- | --- | --- | --- | --- | --- | --- | --- | --- |
|  |  |  |  | *x* | *y* | *z* |  |  |  |
| L |  | Insula | 576 | -48 | 00 | 14 | -5.56 | .001 | < .0001 |
| L | Frontal | Inferior Frontal Gyrus, Precentral Gyrus, Rolandic operculum, Supplementary Motor Area |  |  |  |  |  |  |  |
| L | Parietal | Postcentral Gyrus |  |  |  |  |  |  |  |

*Note*. Second level SCA results: resting-state functional connectivity between the seed region right orbitofrontal cortex and the rest of the brain after 18mg nalmefene compared to placebo (contrast: nalmefene > placebo). Combined voxel-wise-threshold (*p* < .01) and cluster-extent threshold *k* > 576 Voxel, corresponding to *p*FDR < .05; MNI = Montreal Neurological Institute.

## **Fig. S7**

*Reflective System: Resting-State Functional Connectivity with the Seed Region “Right Orbitofrontal Cortex” after 18mg Nalmefene Compared to Placebo*

*
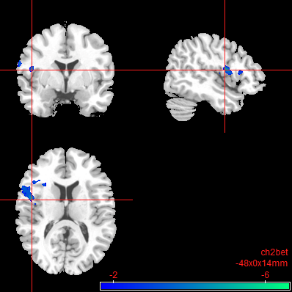
*

*Note.* Brain regions with decreased resting-state functional connectivity between the seed region right orbitofrontal cortex and the rest of the brain after 18mg nalmefene compared to placebo (contrast: nalmefene > placebo). Combined voxel-wise-threshold (*p* < .01) and cluster-extent threshold *k* > 576 Voxel, corresponding to *p*FDR < .05

## **Table S7**

*Default Mode Network: Resting-State Functional Connectivity with the Seed Region “Medial Prefrontal Cortex” after 18mg Nalmefene Compared to Placebo*

| Side | Lobe | Brain Areas | Size | MNI Coordinates | | | t_max_ | Cluster  *p*FDR | Peak  *p*-unc |
| --- | --- | --- | --- | --- | --- | --- | --- | --- | --- |
|  |  |  |  | *x* | *y* | *z* |  |  |  |
| R |  | Insula, Putamen | 80 | 28 | 08 | -10 |  | .0609 | < .0001 |
|  |  |  |  |  |  |  |  |  |  |

*Note*: Second level SCA results: resting-state functional connectivity between the seed region medial Prefrontal Cortex and the rest of the brain after 18mg nalmefene compared to placebo (contrast: nalmefene > placebo). Combined voxel-wise-threshold (*p* < .001) and cluster-extent threshold *k* > 80 Voxel, corresponding to *p*FDR < .05; MNI = Montreal Neurological Institute.

## **Fig. S8**

*Default Mode Network: Resting-State Functional Connectivity with the Seed Region “Medial Prefrontal Cortex” after 18mg Nalmefene Compared to Placebo*


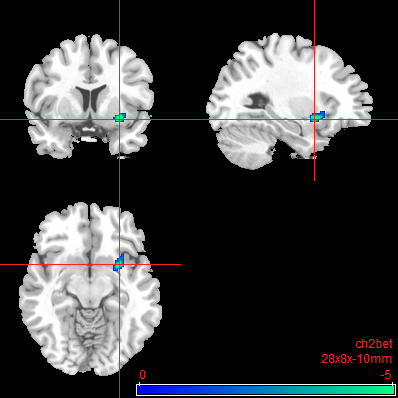


*Note.* Brain regions with decreased resting-state functional connectivity between the seed region medial prefrontal cortex and the rest of the brain after 18mg nalmefene compared to placebo (contrast: nalmefene > placebo). Combined voxel-wise-threshold (*p* < .001) and cluster-extent threshold *k* > 80 Voxel, corresponding to *p*FDR < .05
